# Essential turmeric oils enhance anti-inflammatory efficacy of curcumin in dextran sulfate sodium-induced colitis

**DOI:** 10.1038/s41598-017-00812-6

**Published:** 2017-04-11

**Authors:** Shusuke Toden, Arianne L. Theiss, Xuan Wang, Ajay Goel

**Affiliations:** 1 Center for Gastrointestinal Research, Center for Translational Genomics and Oncology, Baylor Scott & White Research Institute and Charles A Sammons Cancer Center, Baylor Research Institute and Sammons Cancer Center, Baylor University Medical Center, Dallas, Texas USA; 2 Department of Internal Medicine, Division of Gastroenterology, Baylor Scott & White Research Institute and Sammons Cancer Center, Baylor University Medical Center, Dallas, Texas USA; 3 Baylor Institute for Immunology Research, Baylor Scott & White Research Institute, Dallas, Texas 75204 USA

## Abstract

Turmeric has been used as a medicinal herb for thousands of years for treatment of various disorders. Although curcumin is the most studied active constituents of turmeric, accumulating evidence suggests that other components of turmeric have additional anti-inflammatory and anti-tumorigenic properties. Herein, we investigated anti-inflammatory efficacy and associated gene expression alterations of a specific, curcumin preparation containing essential turmeric oils (ETO-curcumin) in comparison to standard curcumin at three specific doses (0, 5, 25 or 50 mg/kg), in an animal model of dextran sodium sulfate (DSS)-induced colitis. The present study showed that both ETO and standard curcumin treatments provided protection against DSS-induced inflammation. However, ETO-curcumin improved disease ﻿activity index (DAI) dose-dependently, while the anti-inflammatory efficacy of standard curcumin remained constant, suggesting that ETO-curcumin may provide superior anti-inflammatory efficacy compared to standard curcumin. Gene expression analysis revealed that anti-inflammatory cytokines including IL-10 and IL-11 as well as FOXP3 were upregulated in the colon by ETO-curcumin. Collectively, these findings suggest that the combined treatment of curcumin and essential turmeric oils provides superior protection from DSS-induced colitis than curcumin alone, highlighting the anti-inflammatory potential of turmeric.

## Introduction

Turmeric has been used traditionally as a medicinal herb in India and South East Asia for thousands of years for various illnesses including biliary disorders, anorexia, coryza, cough, hepatic and rheumatic ailments and a variety of other chronic inflammatory diseases. In particular, curcumin, the active principle extracted from the dried rhizomes of *Curcuma longa* (or turmeric) is perhaps one of most studied natural compounds within the context of complementary medicine. In addition to its well-established anti-inflammatory properties, clinical studies in the recent years have highlighted its therapeutic efficacy in a variety of diseases including arthritis and depression^1, 2^, as well as its potent anti-tumorigenic potential^3–6^. Interestingly, growing body of data also indicate that in addition to curcumin, other constituents of turmeric, primarily essential turmeric oils (ETO) comprising of aromatic-tumerones (ar-tumerones), α-turmerones, β-turmerones, α-santalene and aromatic curcumene, also possess significant anti-inflammatory and anti-oxidant properties^7–9^. One of curcumin’s potential limitations is that it is poorly absorbed following ingestion; hence there has been a tremendous interest in developing strategies to enhance its absorption and systemic bioavailability. In this context, previous studies have demonstrated that administration of curcumin complexed with essential turmeric oils (ETO-curcumin) enhanced its bioavailability in circulation by 7–10 fold compared to standard curcumin, which subsequently lead to significantly improved bioactivity^10, 11^. The ETO-curcumin has been shown to exert superior anti-tumorigenic effects by permitting differentiation of cancer stem cells, reversing epithelial-to-mesenchymal transition and by enhancing the efficacy of chemotherapeutic agents such as 5-fluorouracil in *in vitro* and pre-clinical studies^12–14^. Although these data indicate that ETO-curcumin appears to have higher bioactivity, no studies have directly investigated the bioactivity of ETO-curcumin in comparison to standard curcumin and the underlying anti-inflammatory mechanisms.

Ulcerative colitis (UC) is an inflammatory disorder which affects the entire colorectum, and is one of the two major forms of inflammatory bowel diseases (IBD) along with Crohn’s disease (CD). Ulcerative colitis remains one of the most difficult gastrointestinal diseases to manage due to lack of definitive therapies^15^. While anti-tumor necrosis factor alpha (TNF-α) antibodies appear to be moderately effective in clinical management of CD and UC, not all patients respond to these antibodies, and these regimens often associate with severe side-effects^16^. Emerging evidence suggests that dysregulation of inflammatory transcription factors such as NF-κB and signal transducers and activators of transcription (STAT) could be involved in pathogenesis of UC^17–19^. Based upon these findings, several molecular inhibitors are currently being developed to target these inflammatory pathways. It is noteworthy to mention that the effectiveness of curcumin has been consistently demonstrated in preclinical models of colitis as well as in patients with UC^20–22^, through down-regulation of both NF-κB and STAT pathways^23–26^. Furthermore, a recent randomized, multinational clinical study demonstrated that combined treatment with curcumin and mesalamine resulted in remission in 54% of patients with colitis, while none of the patients achieved clinical remission in the control, untreated group^27^. Considering that curcumin is a readily available, safe and a cost-effective botanical, there is a growing interest in exploring its clinical efficacy individually or as an adjunctive treatment for managing and/or treating UC and subsequently improving the overall quality of life for patients with this inflammatory disease. Although curcumin is a well-established anti-inflammatory agent, the exact mechanism(s) by which it attenuates inflammatory burden in diseases such as UC remains unclear. Herein, we demonstrated that curcumin exhibited significant anti-inflammatory effects in a mouse model of dextran sodium sulfate (DSS)-induced colitis. In particular, a unique formulation of curcumin containing essential turmeric oils (ETO-curcumin) displayed greater anti-inflammatory efficacy compared to the standard curcumin, as evidenced by the reduced disease activity index (DAI) and changes in gene expression alterations of the inflammation-related genes, highlighting that ETO enhances the anti-inflammatory efficacy of curcumin.

## Results

### Curcumin exerts anti-inflammatory effects in DSS-induced colitis

**Figure 1:**
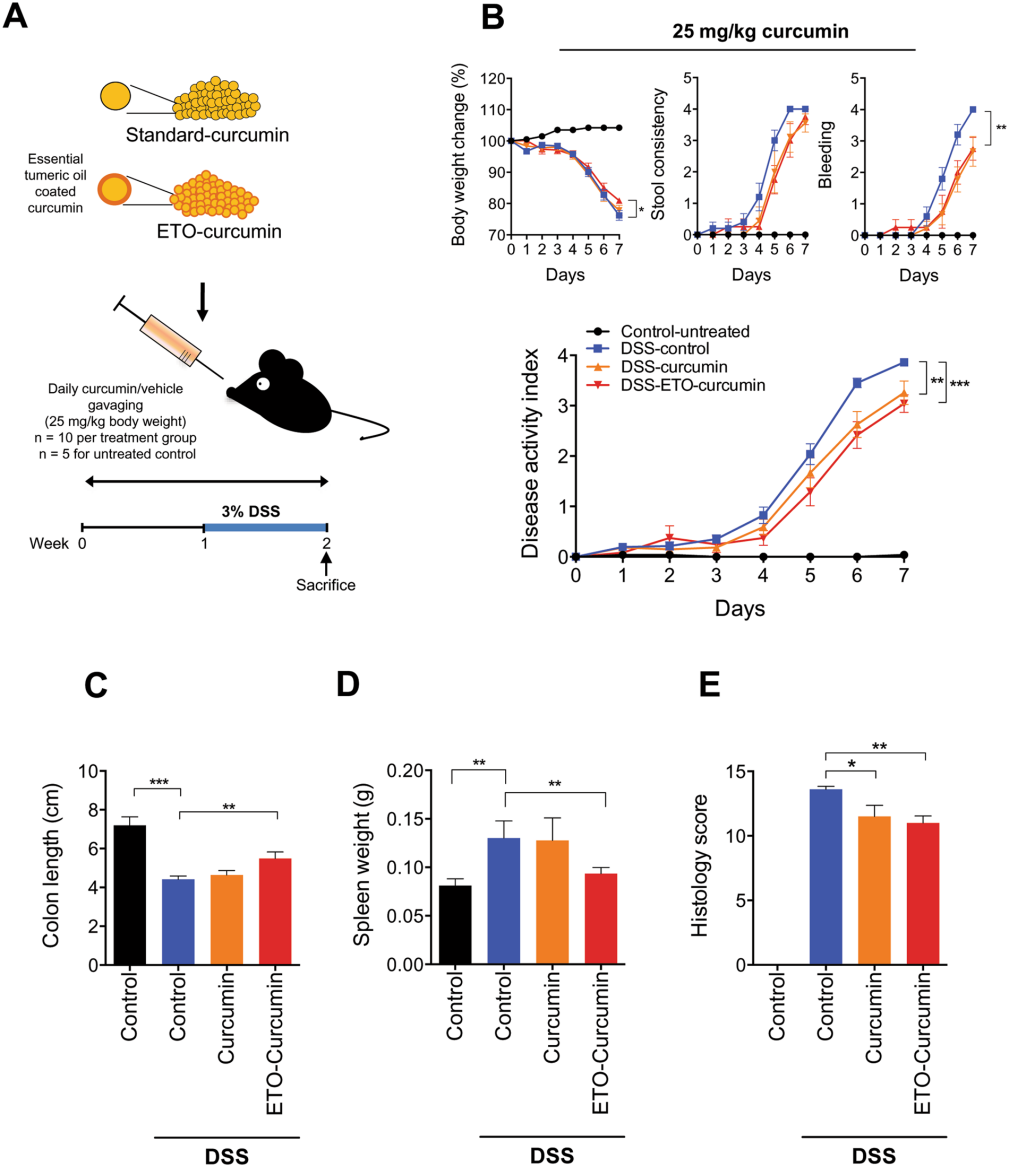
Curcumin attenuates DSS-induced inflammation at 25 mg/kg body weight. (**A**) Graphical representation of curcumin treatment strategy. (**B**) Changes in individual categories of disease activity index (﻿DAI) (top): body weight changes, stool consistency, and stool bleeding (left-right). Changes in DAI (bottom). (**C**) Colon length, (**D**) Spleen weight and (**E**) Histology score on day 14. *p < 0.05, **p < 0.01, ***p < 0.001.

Since curcumin is a well-established botanical with anti-inflammatory properties, we wanted to determine whether presence of essential turmeric oils have any additional bioactivity, and hence, we sought to test the differences in anti-inflammatory efficacy between ETO- and standard curcumin. We first tested the efficacy of both types of curcumin at a relatively low treatment dose of 25 mg/kg body weight. In order for animals to be acclimatized to gavaging, we pretreated mice with curcumin for one week, followed by addition of 3% DSS in their drinking water to induce colitis (Fig. 1A). Based on the DAI values, both ETO-curcumin and standard curcumin-fed animals had less severe colitis from DSS treatment compared to the control animals, as early as day 6 (*p* < 0.001 and *p* < 0.01 respectively; Fig. 1B). Similarly, on day 7 of DSS treatment, both curcumin treatments showed reduction in DAI compared to DSS-control group, indicating their ability to attenuate inflammation-mediated colitis. Analyzing individual measures of DAI, ETO-curcumin treated mice maintained superior DAI parameters compared to the DSS control group, in particular the body weight loss category. None of the curcumin treatments altered stool consistency, but both were effective in reducing fecal bleeding compared to DSS-control mice (*p* < 0.01). Since colon length is another well-established indicator of DSS-induced colitis, we examined the length of colon after 7 days of DSS treatment. As expected, DSS treatment resulted in colon shortening, while ETO-curcumin, but not standard curcumin, significantly prevented such colon shortening (*p* < 0.01; Fig. 1C). Similarly, enlargement of spleen is another indicator of immune response to inflammation. Spleen weight in ETO-curcumin treated mice, but not standard curcumin, was lower compared to DSS-treated mice (*p* < 0.01), further confirming its anti-inflammatory effects (Fig. 1D). In addition, both ETO- and standard curcumin treated mice had lower histological scores than DSS-controls (*p* < 0.01 and *p* < 0.05 respectively; Fig. 1E). Taken together, while several DAI parameters showed an improved trend toward ETO-curcumin having superior anti-inflammatory efficacy than standard curcumin, our results demonstrated that both curcumin preparations were effective in mitigating DSS-induced colitis.

### ETO-curcumin exhibited superior anti-inflammatory effects over standard-curcumin

**Figure 2:**
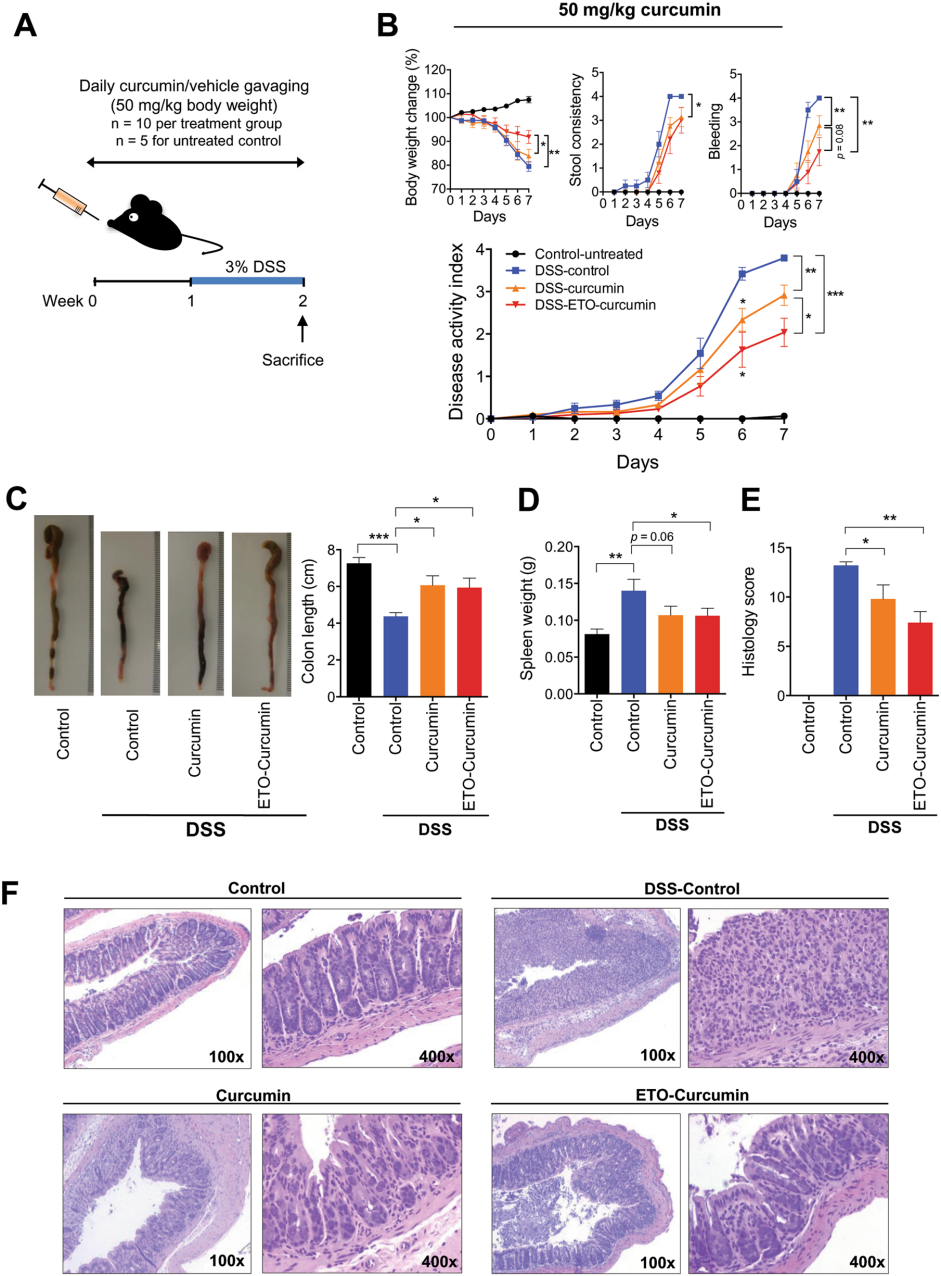
ETO-curcumin exerts superior anti-inflammatory effects compared to standard curcumin on DSS-induced inflammation at 50 mg/kg body weight. (**A**) Graphical representation of curcumin treatment strategy. (**B**) Changes in individual categories of disease activity index ﻿(DAI) (top), body weight changes, stool consistency and stool bleeding (left-right). Changes in DAI (bottom). (**C**) Representative image of colons (left) and average colon length (right). (**D**) Spleen weight and (**E**) Histology score on day 14. (**F**) Representative haematoxylin and easin (H&E) staining of large intestine on day 14. 100x magnification (left) and 400x magnification (right). *p < 0.05, **p < 0.01, ***p < 0.001.

In order to determine whether ETO-curcumin has a superior anti-inflammatory efficacy compared to standard curcumin, we doubled the treatment dose (50 mg/kg body weight) to re-evaluate the effects of these two curcumin types (Fig. 2A). Moreover, this dose has previously been established as an optimal dose for standard curcumin for DSS-induced rodent study^20^. During the first three days of treatment, no differences in DAI were observed between DSS-treated groups regardless of curcumin treatment (Fig. 2B). However, from day 4 of DSS treatment, ETO-curcumin treated mice displayed significant attenuation of DAI compared to DSS-control group, while animals treated with standard curcumin showed a delayed attenuation in DAI. Furthermore, ETO-curcumin showed lower DAI at day 7 of DSS treatment compared to both DSS control and standard curcumin treated groups (*p* < 0.001 and *p* < 0.05) indicating that ETO-curcumin was significantly more effective in attenuating DSS-induced colitis than standard curcumin. In order to determine which specific DAI parameters contributed to ETO-curcumin’s superior anti-inflammatory efficacy over standard curcumin, we further assessed each DAI criterion individually. While both ETO- and standard curcumin formulations were equally effective for maintaining consistency in feces (both *p* < 0.05 compared to DSS-control), ETO-curcumin-treated animals showed superior attenuation in DSS-induced body weight loss compared to standard curcumin (*p* < 0.05; Fig. 2B). In addition, we noted a statistical trend indicating that ETO-curcumin treatment resulted in less severe DSS-induced intestinal bleeding (*p* < 0.08) compared to standard curcumin treatment group. As expected, DSS treatment resulted in shrinkage of the colon, while both curcumin treatments attenuated shortening of the large intestine (both *p* < 0.05; Fig. 2C). Although ETO-curcumin treated mice demonstrated reduced spleen weight compared to DSS-treated group (*p* < 0.05), standard curcumin treated group only showed a trend in spleen weight reduction (*p* = 0.06; Fig. 2D). Consistent with our DAI measurements, histological evaluation showed that both ETO- and standard curcumin significantly attenuated DSS-induced colitis (*p* < 0.01 and *p* < 0.05 respectively; Fig. 2E and F). Collectively, these data indicate that ETO-curcumin resulted in superior anti-inflammatory effects compared to standard curcumin, suggesting that the presence of ETO may synergistically enhance the bioactivity of curcumin in the colon.

Next, to determine whether the superior anti-inflammatory efficacy of ETO-curcumin was due to combined effects of ETO and curcumin or as a result of independent contribution of ETO, we repeated these experiments with the addition of an ETO alone group. Consistent with our initial experiments, once again we observed significant attenuation of DAI for both curcumin and ETO-curcumin treatment groups (Supplementary Fig. 1). Furthermore, to our surprise, ETO gavaged animals showed no difference in DAI compared to that of DSS-control group, suggesting that anti-inflammatory effects of ETO-alone are minimal (Supplementary Fig. 1). Therefore, our data indicates that ETO enhanced the anti-inflammatory efficacy of curcumin.

### ETO-curcumin enhances anti-inflammatory effects in a dose-dependent manner

**Figure 3:**
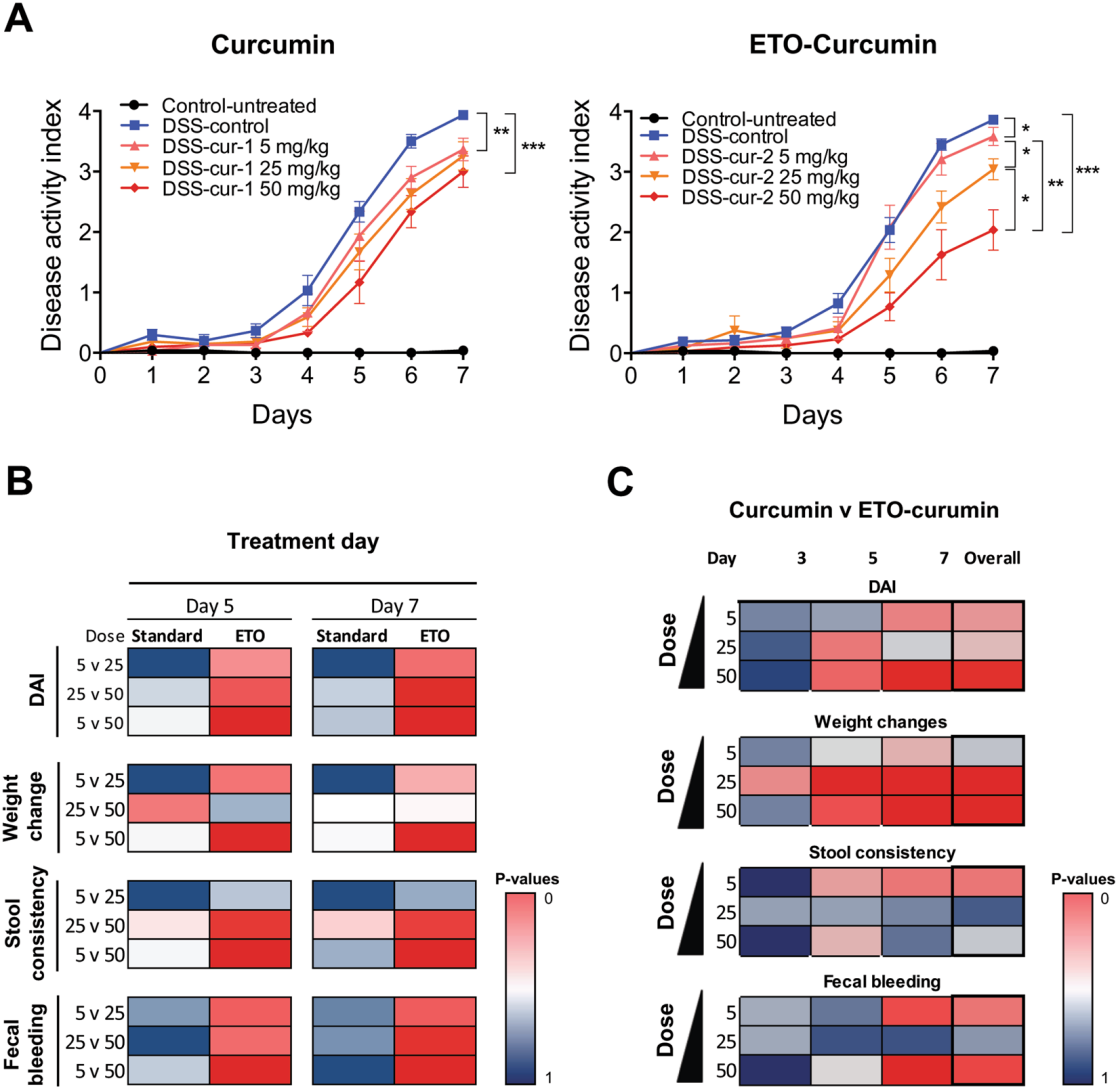
Dose-dependent comparison between standard curcumin and ETO-curcumin on disease activity index (DAI). (**A**) DAI of standard curcumin (left) and ETO-curcumin (right) according to their doses. (**B**) Graphical representation of comparison of doses within the treatment group at DSS treatment days 5 and 7 for DAI and DAI associated parameters. (**C**) Graphical representation of comparison between standard curcumin and ETO-curcumin at individual doses (5, 25, 50 mg/kg) on DSS treatment days 3, 5, 7 and cumulative comparsion. For both panel B and C red cells represent significant difference between groups, while blue represents similar values.

We next used a mathematical model, “linear mixed model”, to further evaluate the efficacy of ETO and standard curcumin. We assessed the dose-related effects over three doses - 5, 25 and 50 mg/kg body weight (5 mg/kg shown in Supplementary Fig. 2). The combined DAI across three doses for both types of curcumin are shown in Fig. 3A. Considering that all treatment doses for both curcumin extracts demonstrated significant attenuation in DAI, we first examined whether these protective effects are dose-dependent. To evaluate this hypothesis, we compared DAI and its associated sub-categories across all doses at day 5 and 7 of DSS treatment and graphically represented dose-associated effectiveness of each curcumin type (Fig. 3B and Supplementary Table 1). We color coded each cell according to its effectiveness with red representing the highest improvement in the effectiveness between doses, while blue representing no difference. Surprisingly, within the standard curcumin group, we observed no statistical difference between doses for DAI, weight change, stool consistency and fecal bleeding at both days 5 and 7. These data indicate that although standard curcumin clearly reduces DAI, the anti-inflammatory efficacy of standard curcumin was not dose-dependent (Fig. 3B and Supplementary Table 1). In contrast, ETO-curcumin demonstrated a dose-dependent improvement in efficacy at both days 5 and 7. Significant improvements were observed for DAI (*p* < 0.001), weight change (*p* < 0.001) and fecal bleeding (*p* = 0.001) when 50 mg/kg treatment group was compared to 5 mg/kg treatment group at both days 5 and 7 (Fig. 3B and Supplementary Table 1). Furthermore, the comparison between 25 mg/kg and 50 mg/kg showed improvement in DAI (*p* < 0.05) for ETO-curcumin in both days 5 and 7 compared to standard curcumin. Collectively these results suggest that ETO-curcumin enhanced anti-inflammatory efficacy in a dose-dependent manner in a DSS-induced colitis animal model, while standard curcumin appeared to show no dose-associated improvement in DAI, at least for the doses that we evaluated.

Next, we comprehensively analyzed the anti-inflammatory efficacies between ETO- and standard curcumin at each dose (5, 25 and 50 mg/kg) at specific time points (days 3, 5 and 7). Once again we represented our data graphically to illustrate time and dose-dependent dynamics of two curcumin types (Fig. 3C). Although differences between ETO and standard curcumin were small at DSS treatment day 5, the difference in DAI associated parameters between ETO- and standard curcumin treatments at day 7 increased substantially. In particular, we found statistically significant differences between ETO- and standard curcumin for body weight changes during days 5 and 7 for 25 and 50 mg/kg doses (*p* < 0.001). Within the group with highest treatment dose (50 mg/kg), ETO-curcumin demonstrated significant improvements in DAI, weight change, and fecal bleeding at day 7 compared to standard curcumin (all *p* < 0.05: Fig. 3C and Supplementary Table 2). Collectively, these data represent significant time-dependent shift in effectiveness between ETO- and standard curcumin for DAI suggesting that ETO-curcumin exerted superior anti-inflammatory effects compared to standard curcumin.

### Curcumin alters gene expression profiles of inflammation-associated genes

**Figure 4:**
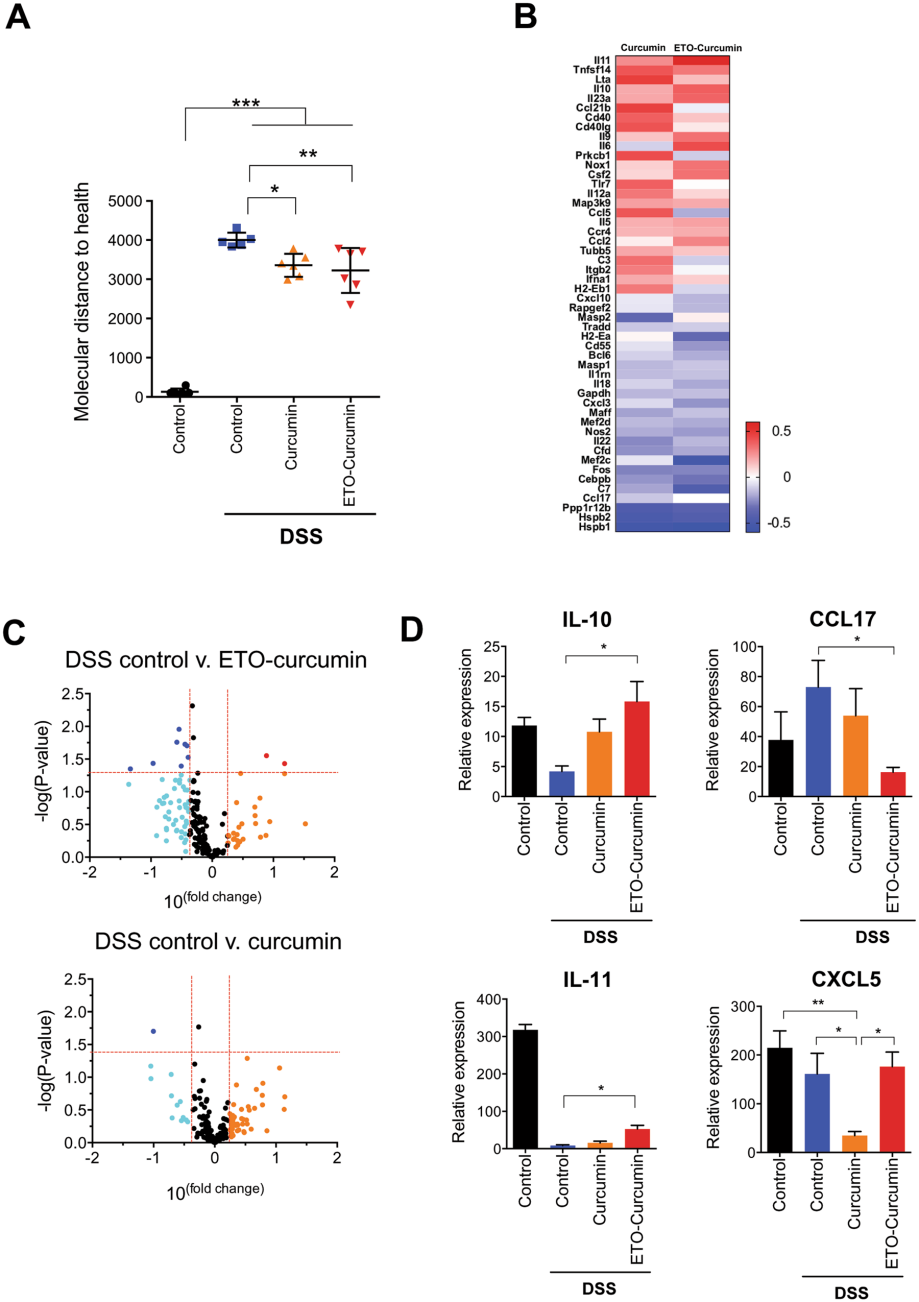
Curcumin alters gene expression of inflammation associated genes (**A**) Treatment groups represented using molecular distance to health (MDTH) (**B**) Heat map of DSS-control, ETO-curcumin and curcumin normalized to untreated mice (**C**) Volcano plot comparison between control group and ETO-curcumin﻿ treated group (top) control group and curcumin treated group (bottom) (**D**) Expression levels of IL-10, IL-11, CCL17, CXCL5 determined by nanoString.

To clarify the anti-inflammatory mechanism of curcumin, we investigated the gene expression profiles of inflammation-associated genes from tissues collected from colon in animals treated with 50 mg/kg dose using a Nanostring platform. As expected, there were significant alterations in the expression profiles of inflammatory genes in both curcumin treated vs. DSS-control groups (Fig. 4A). Since the overall gene expression profile of all DSS treatment groups appear to be relatively similar, we further analyzed our data using a mathematical estimation called “molecular distance to health” (MDTH) to determine whether there is a significant difference in overall gene expression profiles between DSS-control and curcumin groups. MDTH is a mathematical estimation which calculates overall shift in expression of genes between treatment groups according to their deviations in gene expression signature^28^. Intriguingly, MDTH analysis showed considerable shift in overall gene expression profile when animals treated with DSS were compared to non-DSS treatment control group (*p* < 0.001; Fig. 4B). However, both curcumin treatment groups significantly shifted MDTH towards non-DSS control group when compared to DSS-control group (ETO-curcumin, *p* < 0.01 and standard curcumin *p* < 0.05), indicating that curcumin treatments attenuated overall shift towards inflammatory molecular signature of control-DSS group (Fig. 4B). Due to relatively large variation in MDTH values within ETO-curcumin treated group, we were unable to detect difference between ETO- and standard curcumin treatments using MDTH.

Next, we examined specific genes which were altered with curcumin treatment by first identifying differentially expressed genes between ETO- and standard curcumin against DSS-controls with greater than 1.5 fold change. A total of 50 differentially expressed genes were identified, suggesting that both ETO and standard curcumin altered common anti-inflammatory molecular pathways (Fig. 4C). We further interrogated the data to identify most differentially expressed genes for both ETO-curcumin and standard curcumin in comparison to DSS-controls and represented the data in volcano plots (Fig. 4D). We initially identified 10 such genes, which included 2 upregulated (IL10 and IL11) and 8 downregulated (CCL17, HSPB1, MEF2C, C7, HSPB2, PPP1R12B, H2-EA and CEBPB) genes for ETO-curcumin group, and one downregulated gene for the standard curcumin group, CXCL5, (Fig. 4D). Based on mechanistic findings of previous studies^29–31^, we decided to focus on two most highly upregulated genes for ETO-curcumin as well as a downregulated gene (CCL17) and the lone differentially expressed gene for standard-curcumin (CXCL5) (Fig. 4E).

### ETO-curcumin attenuates inflammation through upregulation of anti-inflammatory cytokines

**Figure 5:**
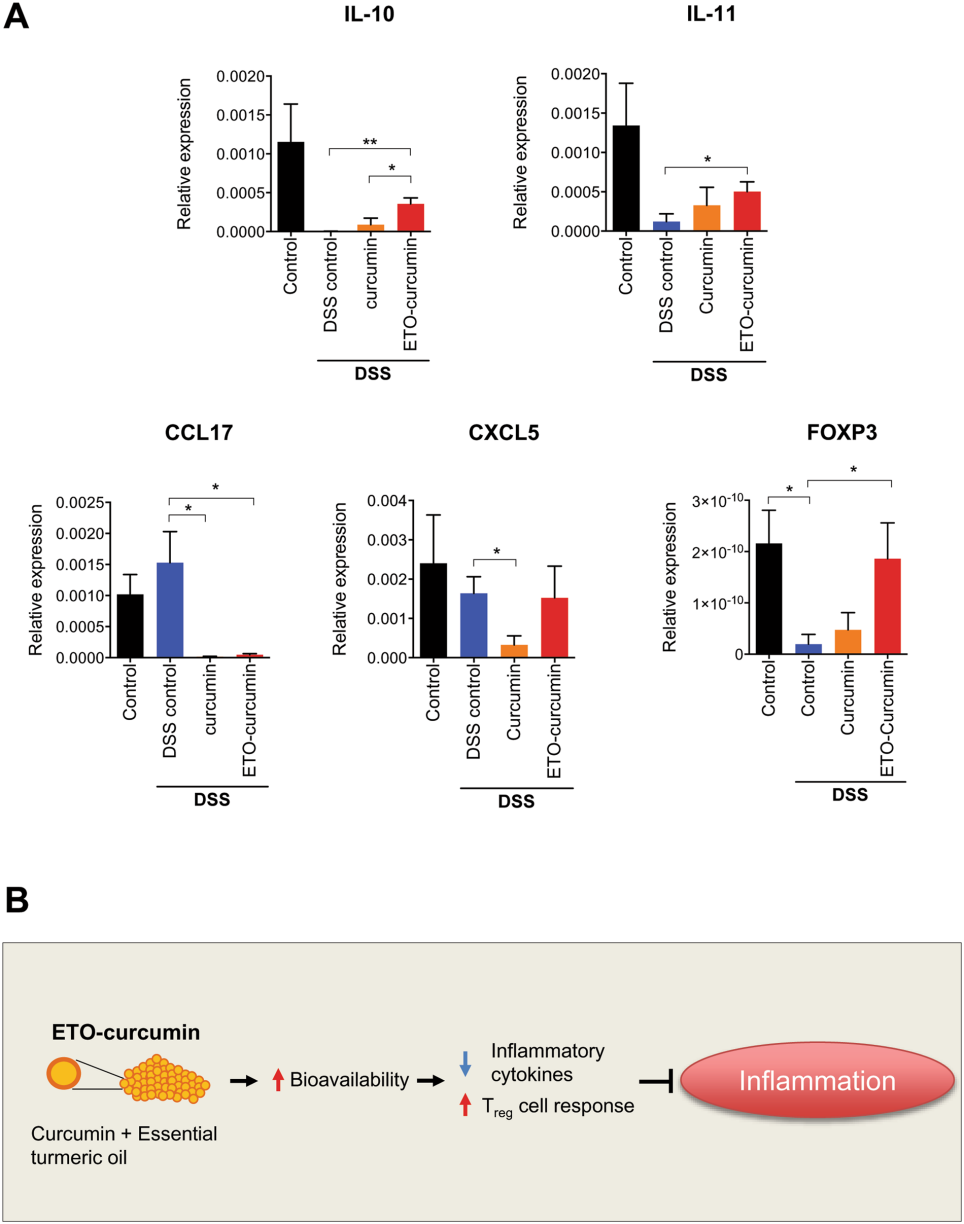
Curcumin suppresses inflammation through modulation of inflammation associated genes. (**A**) Gene expression of IL-10, IL-11, CCL17, CXCL5 and FOXP3 normalized to 18S (**B**) Schematic representation of how ETO-curcumin exerts its anti-inflammatory effects.

In order to validate the genes identified by Nanostring array, we used qRT-PCR to assess the gene expression of the identified genes from the colonic tissue samples (Fig. 5). Intriguingly, these two anti-inflammatory cytokines identified by Nanostring array, IL-10 and IL-11, are well-known anti-inflammatory cytokines. IL-10 is a well-established anti-inflammatory cytokine essential for the maintenance of the epithelial layer homeostasis and minimizes damages obtained from events such as infection^29^. Similarly IL-11 is another cytokine with anti-inflammatory properties manifested through suppression of TNF-α and IL-1^30^. Confirming our Nanostring array data, the expression of anti-inflammatory cytokines, IL-10 and IL-11, was upregulated by ETO-curcumin compared to DSS-control (*p* < 0.01 and *p* < 0.05 respectively). We next determined whether upregulation of IL-10 is caused by upregulation of FOXP3, a master regulatory transcription factor of regulatory T-cells (T_reg_ cells), in DSS-induced colitis^32^. T_reg_ cells have been shown to suppress immune responses in humans and inhibit colitis through IL-10 production^33^. As expected DSS treatment resulted in significant downregulation of FOXP3 expression (*p* < 0.05), but ETO-curcumin was able to attenuate DSS-induced FOXP3 downregulation (*p* < 0.05). Our data suggest that ETO-curcumin may stimulate the T_reg_ cell production and subsequently upregulate IL-10. In contrast, we confirmed that CCL17, a chemokine involved in induction of intestinal inflammation^31^, was suppressed by both standard curcumin and ETO-curcumin treatments. Interestingly, CXCL5 expression was downregulated in standard curcumin-treated mice, while the effect was not seen in the ETO-curcumin-treated group, suggesting that standard curcumin may exert anti-inflammatory effects through different mechanisms. Collectively, these data indicate that oral administration of curcumin protects against DSS-induced colitis through modulation of immune response.

## Discussion

In contrast to many south eastern Asian countries, consumption of turmeric as a dietary spice is generally uncommon in the western world. Nonetheless, in view of growing scientific evidence for the health benefits of curcumin, the active principle component present in the turmeric, curcumin supplements are becoming more widely accepted and popularized in the western nations. However, a question remains whether commercially-available curcumin extracts claiming enhanced bioavailability function comparably and whether curcumin supplementation is an equally effective approach as consuming this phytomedicine through dietary turmeric. The present study was undertaken to address some of these questions, and we deliberately chose to compare the anti-inflammatory effects of standard curcumin with a high-absorption curcumin, ETO-curcumin, in a well-established animal model of colitis. In this study we made several key observations. First, we confirmed anti-inflammatory properties of curcumin on the disease activity index in an animal model of DSS-induced colitis. Second, our data indicates that ETO-curcumin displayed a superior anti-inflammatory efficacy than that of standard curcumin and the subsequent mathematical analysis revealed that the ETO-curcumin associated anti-inflammatory effects were particularly pronounced at higher doses. Third, further investigation of the mechanisms by which ETO-curcumin exerted anti-inflammatory effects showed that ETO-curcumin upregulated anti-inflammatory cytokines including IL-10 and IL-11 and suppressed the chemokine CCL17.

Despite the majority of turmeric-related research has primarily focused on curcumin, less studied components of turmeric such as ETO, has been shown to have significant anti-inflammatory property. In particular, ar-turmerone has shown to possess potent anti-inflammatory, anti-oxidative and anti-platelet properties^8, 9^. Furthermore, clinical studies have demonstrated that regular ETO-curcumin consumption improved diseases including arthritis, depression and Alzheimer’s disease^1, 2, 34^. Although currently there is no study conducted on comparing anti-inflammatory properties of ETO and curcumin, ETO has been shown to exert more potent antifungal activity than curcumin^35^. Our data indicated that ETO-curcumin exerted superior anti-inflammatory effects than standard curcumin in DSS-induced mouse model of colitis and that the ETO enhances bioactivity of curcumin. Consistent with our data, combined treatment of curcumin and ar-turmerone has been shown to reduce adenoma multiplicity in DSS-dimethylhydrazine-induced inflammation associated model of CRC^36^. Although we showed that ETO-curcumin had overall superior anti-inflammatory efficacy than that of standard curcumin, we observed that ETO was less effective at lower doses. One potential reason for ETO-curcumin not being able to exert superior anti-inflammatory effects compared to the standard curcumin treatment group could be low ETO dose in these treatment groups. Furthermore, we demonstrate that mice gavaged with ETO alone showed no influence on DAI, suggesting that ETO contributed to enhanced activity of curcumin rather than acting as a potent anti-inflammatory agent on its own. Although our data indicates that the enhancement of bioactivity of ETO-curcumin is not exclusively related to bioavailability of curcumin, it is important to note that absorption of curcumin has been shown to increase significantly with the presence of lipophilic turmerones^37^.

In the present study, we investigated a range of curcumin doses which represented equivalent concentration of typical human consumption. Therefore, we chose our highest gavaging dose as 50 mg/kg body weight based on a previous study which showed that curcumin exerted anti-inflammatory effects at this dose in a DSS-induced colitis model^20^. According to the animal-to-human dose conversion formula using intestinal surface area, a mouse gavaged with 50 mg/kg body weight curcumin is equivalent to an individual with 70 kg weight consuming 283 mg curcumin^38^. While this calculation is relatively crude, this dose is still significantly less than a typical commercially available curcumin capsule which generally contains approximately 500 mg of curcumin. We confirmed that curcumin at 50 mg/kg resulted in significant attenuation of DSS-induced colitis and also demonstrated that curcumin at lower doses also attenuated DSS-induced colitis. This is of significant importance as several previous clinical studies have used doses exceeding 2 g per day which required multiple daily intakes^39–41^. While extrapolating animal data to human is difficult, it is important to maintain the doses used in the animal study as close as possible to clinical circumstances.

The present study also assessed gene expression changes of standard curcumin and ETO-curcumin in a panel of inflammation-associated genes. We identified anti-inflammatory cytokines, IL-10 and IL-11, were differentially expressed in ETO-curcumin compared to DSS-control. IL-10 is a well-established anti-inflammatory gene which is dysregulated in inflammatory disease and curcumin has been shown previously to stimulate promoter activity of IL-10 gene^42^. We have demonstrated that curcumin, ETO-curcumin in particular, elevated IL-10 expression in the colonic tissue indicating that ETO-curcumin exerts its anti-inflammatory effects through upregulation of anti-inflammatory cytokines. One of the IL-10 stimulatory mechanisms is T_reg_ cells, which can be assessed through the expression of FOXP3^43^. We demonstrated that FOXP3 expression was suppressed by DSS-induced colitis, but intriguingly curcumin treatments, ETO-curcumin in particular, reversed this suppression. While curcumin has been shown to stimulate T_reg_ cells in an animal model of inflammation^44^, our study indicates that ETO-curcumin was more effective in exerting T_reg_ cells mediated anti-inflammatory immune response. In addition, we identified inflammation-associated genes; CCL17 and CXCL5 were modulated by curcumin treatments. CCL17 is a chemokine which is required for induction of intestinal inflammation and counteracts T_reg_ cells mediated protection from colitis^31^. While exact mechanism by which curcumin modulate this complex immune response requires further investigation, our data suggests that curcumin appears to have profound effects on immune response to inflammation. Although curcumin has been shown in modulate several anti-inflammatory pathways, the present study assessed the anti-inflammatory molecular profile at day 7 of DSS treatment. At this stage colitis was already severe for the majority of animals and this was reflected in the overall gene expression changes represented by the molecular distance to health (MDTH) assessment. Therefore, this could be the reason that only relatively small numbers of genes were differentially expressed in both standard curcumin and ETO-curcumin groups. Nevertheless, our data show that ETO-curcumin altered overall anti-inflammatory gene expression profile greater than that of standard curcumin reflecting its anti-inflammatory efficacy seen in DAI. Based on the findings of the present study, we illustrated potential mechanisms of ETO-curcumin in Fig. 5B. ETO-curcumin could exert more potent anti-inflammatory effect due to enhanced bioavailability, superior modulation of inflammatory cytokines and subsequent stimulation of T_reg_ cells.

In conclusion, we demonstrated that both standard curcumin and ETO-curcumin attenuated DSS-induced colitis, while our data indicated that ETO-curcumin was significantly superior in reducing the DAI and inflammatory burden in DSS-induced colitis animal model. The combination of curcumin with essential turmeric oils appear to exert higher bioactivity than stand-alone curcumin highlighting the importance of other components of turmeric for treatment of large intestinal diseases.

## Methods

### Animals and Experimental Protocol

Male C57BL/6 mice (5 weeks old) were purchased from Harlan Laboratories (Houston, TX) and kept under controlled conditions of light (12 hr light and dark cycles). All animals were fed Harlan Teklad Irradiated Global 19% Protein Extruded Diet (Harlan Laboratories) *ad libitum* and provided free access to water. The animal protocol was approved by the Institutional Animal Care and Use Committee of the Baylor Scott & White Research Institute and conducted strictly in accordance to the National Institute of Health Guide for the Care and Use of Laboratory Animals (8^th^ Edition Institute for Laboratory Animal Research). After 1 week of acclimatization, mice were gavaged with curcumin (ETO-curcumin or standard curcumin) daily. ETO-curcumin (BCM-95) and the same batch of curcumin and ETO used to formulate ETO-curcumin (standard curcumin; n = 10 each group) were provided by Dolcas Biotech (Chester, NJ). Both ETO-curcumin and the standard curcumin were fed to animals at a dose range of 5, 25 and 50 mg/kg. 50 mg/kg ETO treatment group was gavaged with 7.5 mg/kg ETO (equivalent to 15% ETO which is equal to the composition of ETO in ETO-curcumin). All curcumin extracts were dissolved in 1% methyl cellulose (Sigma-Aldrich, St. Louis, MO) in water, while the non-treatment control groups were gavaged with water containing equivalent concentration of methyl cellulose (n = 5). Following 7 days of pre-treatment period, chronic colitis was induced by administration of DSS (3% w/v MW: 36,000~50,000 Colitis grade MP Biomedicals, Santa Ana, CA) dissolved in drinking water. All mice were sacrificed under anesthesia 7 days after DSS treatment and the sections of large intestine were collected in RNAlater and subsequently stored at −80 °C.

### Evaluation of Colitis

The severity of colitis was assessed daily after DSS treatment using disease activity index (DAI) by evaluating changes in body weight, stool consistency and fecal blood as described previously^45^. For fecal blood analysis, presence of visible blood in feces and fecal bleeding were designated with a DAI classification score of 2 and 4 respectively. Colonic tissues including 2 cm long distal section of large intestine and 3 cm long section from the rectum were fixed in 10% buffered formalin, paraffin-embedded, sectioned and stained with haematoxylin and eosin (H&E). Histological scores of H&E-stained tissues were conducted blindly according to the criteria described previously^46^.

### Analysis of Colonic Inflammatory Markers

Colon tissues, approximately 2 cm long, and 1 cm away from the rectum were rinsed thoroughly by PBS and stored in RNAlater (Sigma-Aldrich, St. Louis, MO) at −80 °C. Six randomly selected colonic tissues from 50 mg/kg standard and ETO- curcumin groups as well as their respective non-treatment and DSS-treatment control groups were used for the gene expression analysis. The nCounter GX mouse inflammation kit (Nanostring Technologies, Seattle, WA) was used to determine gene expression alterations in inflammation-associated genes. In brief, RNA extracts from colon tissues were subjected to nCounter RNA sample preparation. Thereafter, 100 ng of RNA was used to hybridize probes at 65 °C for 18 hours, followed by purification and analysis on an nCounter Prep Station and Digital Analyzer. The raw data were normalized using nSolver (Nanostring Technologies) using built-in positive controls and the housekeeping genes were used to normalize and calculate mRNA content. Data were then analyzed by multiple t-test with correction for multiple comparison using Sidak-Bonferroni methods using JMP genomics (SAS, Cary, NC).

### Mathematical Analysis

To evaluate the dose associated-effects of standard curcumin and ETO-curcumin, we used linear mixed model approach to mathematically model correlation across time points within the same animal over a period of time. Response variables assessed by this model included DAI (disease activity index) as well as the three individual DAI parameters (fecal bleeding, stool consistency and body weight change). Explanatory variables incorporated in this model included curcumin type (ETO-curcumin v. standard curcumin), curcumin dosages (5, 25 and 50 mg/kg) and different time points (day 3, 5 and 7). We analyzed dose-dependent effects of each type of curcumin independent of time points, as well as at specific time points (e.g. treatment duration). Furthermore, statistical comparisons between ETO-curcumin and standard curcumin were evaluated at specific doses in both, time-dependent and independent fashion. All analyses were conducted using JMP Genomics (SAS, Cary, NC).

### qRT-PCR Analysis

Total RNA was extracted from colonic tissue samples using the Oligotex Direct mRNA mini kit (Qiagen) following the manufacturer’s instructions. For analysis of the expression of mRNA, total RNA was reverse transcribed to complimentary DNA from 1 µg of total RNA using Advantage RT PCR-kit (Clontech Laboratories Inc., Mountain View, CA). Power SYBR Green (Applied Biosystems, Foster City, CA) real-time PCR was performed using StepOnePlus system (Applied Biosystems). For specific primer sequences refer to Supplementary Table (Supplementary Table 1). All qRT-PCR target gene expression was normalized to the expression of 18S and analyzed using the ΔΔCt method.

### Statistical Methods

All analyses were performed using GraphPad Prism Ver. 6.0 (GraphPad Software Inc. San Diego, CA) and JMP Genomics (SAS, Cary, NC). All data were expressed as mean ± SEM with statistical significance indicated when *p* < 0.05. Statistical comparisons between control and treatment groups were determined using student’s t-test or one-way ANOVA with Tukey’s post-hoc correction.

## Electronic supplementary material

Supplementary methods and figures

## References

[1] Chandran B, Goel A (2012). A randomized, pilot study to assess the efficacy and safety of curcumin in patients with active rheumatoid arthritis. Phytother Res.

[2] Sanmukhani, J. *et al*. Efficacy and Safety of Curcumin in Major Depressive Disorder: A Randomized Controlled Trial. *Phytotherapy research*: PTR, doi:10.1002/ptr.5025 (2013). 10.1002/ptr.5025 23832433

[3] Rao CV, Simi B, Reddy BS (1993). Inhibition by dietary curcumin of azoxymethane-induced ornithine decarboxylase, tyrosine protein kinase, arachidonic acid metabolism and aberrant crypt foci formation in the rat colon. Carcinogenesis.

[4] Pereira MA (1996). Effects of the phytochemicals, curcumin and quercetin, upon azoxymethane-induced colon cancer and 7,12-dimethylbenz[a]anthracene-induced mammary cancer in rats. Carcinogenesis.

[5] Huang MT, Wang ZY, Georgiadis CA, Laskin JD, Conney AH (1992). Inhibitory effects of curcumin on tumor initiation by benzo[a]pyrene and 7,12-dimethylbenz[a]anthracene. Carcinogenesis.

[6] Rao CV, Rivenson A, Simi B, Reddy BS (1995). Chemoprevention of colon carcinogenesis by dietary curcumin, a naturally occurring plant phenolic compound. Cancer Res.

[7] Singh G (2010). Comparative study of chemical composition and antioxidant activity of fresh and dry rhizomes of turmeric (Curcuma longa Linn.). Food Chem Toxicol.

[8] Park SY, Kim YH, Kim Y, Lee SJ (2012). Aromatic-turmerone attenuates invasion and expression of MMP-9 and COX-2 through inhibition of NF-kappaB activation in TPA-induced breast cancer cells. J Cell Biochem.

[9] Lee HS (2006). Antiplatelet property of Curcuma longa L. rhizome-derived ar-turmerone. Bioresour Technol.

[10] Shishu mm (2010). Comparative bioavailability of curcumin, tumeric, and Biocurcumax in traditional vichles using non-everted rat intestinal sac model. J Functional Foods.

[11] Antony B (2008). A Pilot Cross-Over Study to Evaluate Human Oral Bioavailability of BCM-95CG (Biocurcumax), A Novel Bioenhanced Preparation of Curcumin. Indian J Pharm Sci.

[12] Buhrmann C (2014). Curcumin suppresses crosstalk between colon cancer stem cells and stromal fibroblasts in the tumor microenvironment: potential role of EMT. PLoS One.

[13] Shakibaei M (2014). Curcumin chemosensitizes 5-fluorouracil resistant MMR-deficient human colon cancer cells in high density cultures. PLoS One.

[14] Toden S (2015). Curcumin mediates chemosensitization to 5-fluorouracil through miRNA-induced suppression of epithelial-to-mesenchymal transition in chemoresistant colorectal cancer. Carcinogenesis.

[15] Katz JA (2002). Advances in the medical therapy of inflammatory bowel disease. Curr Opin Gastroenterol.

[16] Reddy JG, Loftus EV (2006). Safety of infliximab and other biologic agents in the inflammatory bowel diseases. Gastroenterol Clin North Am.

[17] Papadakis KA, Targan SR (2000). Role of cytokines in the pathogenesis of inflammatory bowel disease. Annu Rev Med.

[18] Mudter J (2005). Activation pattern of signal transducers and activators of transcription (STAT) factors in inflammatory bowel diseases. Am J Gastroenterol.

[19] Stevens C (1992). Tumor necrosis factor-alpha, interleukin-1 beta, and interleukin-6 expression in inflammatory bowel disease. Dig Dis Sci.

[20] Liu L (2013). Curcumin ameliorates dextran sulfate sodium-induced experimental colitis by blocking STAT3 signaling pathway. Int Immunopharmacol.

[21] Salh B (2003). Curcumin attenuates DNB-induced murine colitis. Am J Physiol Gastrointest Liver Physiol.

[22] Singla V (2014). Induction with NCB-02 (curcumin) enema for mild-to-moderate distal ulcerative colitis - a randomized, placebo-controlled, pilot study. J Crohns Colitis.

[23] Kim HY, Park EJ, Joe EH, Jou I (2003). Curcumin suppresses Janus kinase-STAT inflammatory signaling through activation of Src homology 2 domain-containing tyrosine phosphatase 2 in brain microglia. J Immunol.

[24] Bharti AC, Donato N, Aggarwal BB (2003). Curcumin (diferuloylmethane) inhibits constitutive and IL-6-inducible STAT3 phosphorylation in human multiple myeloma cells. J Immunol.

[25] Bharti AC, Donato N, Singh S, Aggarwal BB (2003). Curcumin (diferuloylmethane) down-regulates the constitutive activation of nuclear factor-kappa B and IkappaBalpha kinase in human multiple myeloma cells, leading to suppression of proliferation and induction of apoptosis. Blood.

[26] Singh S, Aggarwal BB (1995). Activation of transcription factor NF-kappa B is suppressed by curcumin (diferuloylmethane) [corrected]. J Biol Chem.

[27] Lang A (2015). Curcumin in Combination With Mesalamine Induces Remission in Patients With Mild-to-Moderate Ulcerative Colitis in a Randomized Controlled Trial. Clin Gastroenterol Hepatol.

[28] Berry MP (2010). An interferon-inducible neutrophil-driven blood transcriptional signature in human tuberculosis. Nature.

[29] Ouyang W, Rutz S, Crellin NK, Valdez PA, Hymowitz SG (2011). Regulation and functions of the IL-10 family of cytokines in inflammation and disease. Annu Rev Immunol.

[30] Walmsley M, Butler DM, Marinova-Mutafchieva L, Feldmann M (1998). An anti-inflammatory role for interleukin-11 in established murine collagen-induced arthritis. Immunology.

[31] Heiseke AF (2012). CCL17 promotes intestinal inflammation in mice and counteracts regulatory T cell-mediated protection from colitis. Gastroenterology.

[32] Zhang L, Zhao Y (2007). The regulation of Foxp3 expression in regulatory CD4(+)CD25(+)T cells: multiple pathways on the road. J Cell Physiol.

[33] Asseman C, Mauze S, Leach MW, Coffman RL, Powrie F (1999). An essential role for interleukin 10 in the function of regulatory T cells that inhibit intestinal inflammation. J Exp Med.

[34] Baum L (2008). Six-month randomized, placebo-controlled, double-blind, pilot clinical trial of curcumin in patients with Alzheimer disease. J Clin Psychopharmacol.

[35] Ferreira FD (2013). Inhibitory effect of the essential oil of Curcuma longa L. and curcumin on aflatoxin production by Aspergillus flavus Link. Food Chem.

[36] Murakami A, Furukawa I, Miyamoto S, Tanaka T, Ohigashi H (2013). Curcumin combined with turmerones, essential oil components of turmeric, abolishes inflammation-associated mouse colon carcinogenesis. BioFactors.

[37] Yue GG (2012). The role of turmerones on curcumin transportation and P-glycoprotein activities in intestinal Caco-2 cells. J Med Food.

[38] Reagan-Shaw S, Nihal M, Ahmad N (2008). Dose translation from animal to human studies revisited. FASEB J.

[39] Irving GR (2013). Prolonged biologically active colonic tissue levels of curcumin achieved after oral administration–a clinical pilot study including assessment of patient acceptability. Cancer Prev Res (Phila).

[40] Carroll RE (2011). Phase IIa clinical trial of curcumin for the prevention of colorectal neoplasia. Cancer Prev Res (Phila).

[41] Golombick T, Diamond TH, Badmaev V, Manoharan A, Ramakrishna R (2009). The potential role of curcumin in patients with monoclonal gammopathy of undefined significance–its effect on paraproteinemia and the urinary N-telopeptide of type I collagen bone turnover marker. Clin Cancer Res.

[42] McCann MJ (2014). The effect of turmeric (Curcuma longa) extract on the functionality of the solute carrier protein 22 A4 (SLC22A4) and interleukin-10 (IL-10) variants associated with inflammatory bowel disease. Nutrients.

[43] Murai M (2009). Interleukin 10 acts on regulatory T cells to maintain expression of the transcription factor Foxp3 and suppressive function in mice with colitis. Nat Immunol.

[44] Bereswill S (2010). Anti-inflammatory effects of resveratrol, curcumin and simvastatin in acute small intestinal inflammation. PLoS One.

[45] Murthy SN (1993). Treatment of dextran sulfate sodium-induced murine colitis by intracolonic cyclosporin. Dig Dis Sci.

[46] ten Hove T (2002). Dichotomal role of inhibition of p38 MAPK with SB 203580 in experimental colitis. Gut.

