# Supplementary material for: Essential turmeric oils enhance anti-inflammatory efficacy of curcumin in dextran sulfate sodium-induced colitis
Source: Sci Rep. 2017 Apr 11;7:814. doi: 10.1038/s41598-017-00812-6 (PMC5429743; doi:10.1038/s41598-017-00812-6)

SUPPLEMENTARY INFORMATION

Essential turmeric oils enhance anti-inflammatory efficacy of curcumin in dextran sulfate sodium-induced colitis

Shusuke Toden, Arianne L. Theiss, Xuan Wang and Ajay Goel

Supplementary Table 1: Mathematical comparison of DAI associated parameters between doses for each curcumin treatment

Day 5				
	**Dose comparison**	**P-values**	**Dose comparison**	**P-values**
	**Standard**		**ETO**	
**DAI**	5 v 25	0.764	5 v 25	0.056
	25 v 50	0.248	25 v 50	**0.033**
	5 v 50	0.143	5 v 50	**0.0001**
**Weight change**	5 v 25	0.556	5 v 25	**0.022**
	25 v 50	**0.023**	25 v 50	0.256
	5 v 50	0.071	5 v 50	**0.0007**
**Stool consistency**	5 v 25	0.872	5 v 25	0.477
	25 v 50	0.267	25 v 50	0.058
	5 v 50	0.325	5 v 50	**0.01**
**Fecal bleeding**	5 v 25	0.57	5 v 25	0.099
	25 v 50	0.804	25 v 50	0.113
	5 v 50	0.42	5 v 50	**0.002**

Day 7				
	**Dose comparison**	**P-values**	**Dose comparison**	**P-values**
	**Standard**		**ETO**	
**DAI**	5 v 25	0.937	5 v 25	0.13
	25 v 50	0.463	25 v 50	**0.027**
	5 v 50	0.499	5 v 50	**0.0003**
**Weight change**	5 v 25	0.848	5 v 25	0.05
	25 v 50	0.075	25 v 50	0.071
	5 v 50	0.099	5 v 50	**0.0003**
**Stool consistency**	5 v 25	0.769	5 v 25	0.665
	25 v 50	0.487	25 v 50	0.187
	5 v 50	0.661	5 v 50	0.081
**Fecal bleeding**	5 v 25	0.787	5 v 25	0.165
	25 v 50	0.751	25 v 50	0.061
	5 v 50	0.942	5 v 50	**0.001**

DAI: Disease activity index

Supplementary Table 2: Mathematical comparison between ETO- and standard curcumin in DAI associated parameters

	P-Values			
**Dose (mg/kg)**	**Day 3**	**Day 5**	**Day 7**	**Overall**
	**DAI**			
**5**	0.73	0.657	0.301	0.344
**25**	0.851	0.279	0.529	0.429
**50**	0.921	0.237	**0.043**	0.08
	**Weight changes**			
**5**	0.739	0.505	0.405	0.562
**25**	0.321	**0.002**	**0.003**	**0.0008**
**50**	0.739	0.184	**0.005**	**0.024**
	**Stool consistency**			
**5**	1	0.362	0.274	0.274
**25**	0.656	0.656	0.729	0.849
**50**	1	0.412	0.781	0.549
	**Fecal bleeding**			
**5**	0.624	0.769	0.172	0.271
**25**	0.633	0.873	0.873	0.682
**50**	1	0.493	**0.005**	0.156

DAI: Disease activity index

Supplementary Table 3: Primer lists

Primers		Sequence
FOXP3	(foward)	5’-GGTACACCCAGGAAAGACAG
	(reverse)	5’-ATCCAGGAGATGATCTGCTTG
IL10	(forward)	5’-GGTTGCCAAGCCTTATCGGA
	(reverse)	5’-ACCTGCTCCACTGCCTTGCT
IL-11	(forward)	5’-CTGCAAGCCCGACTGGAA
	(reverse)	5’-AGGCCAGGCGAGACATCA
CCL17	(forward)	5’-CCCATGAAGACCTTCACCTC
	(reverse)	5’-CATCCCTGGAACACTCCACT
CXCL5	(forward)	5’-CTCAGTCATAGCCGCAACCGAGC
	(reverse)	5’-CGCTTCTTTCCACTGCGAGTGC
18S	(forward)	5’-CCCCTCGATGCTCTTAGCTGAGTGT
	(reverse)	5’-CGCCGGTCCAAGAATTTCACCTCT

Supplementary Figure 1: ETO-curcumin exerts superior anti-inflammatory effects compared to standard curcumin and ETO on DSS-induced inflammation at 50 mg/kg body weight A) Graphical representation of curcumin treatment strategy **B**) Changes in individual categories of DAI (top), body weight changes, stool consistency and stool bleeding (left-right). Changes in disease activity index (DAI) (bottom). *= $p < 0.05$, **= $p < 0.01$, ***= $p < 0.001$.

Supplementary Figure 2: Effects of ETO-curcumin and standard curcumin on DSS-induced inflammation at 5 mg/kg body weight daily gavaging A) Graphical representation of curcumin treatment strategy **B**) Changes in individual categories of DAI (top), body weight changes, stool consistency and stool bleeding (left-right). Changes in disease activity index (DAI) (bottom). **C**) Colon length, **D)** Spleen weight and **E**) Histology score on day 7. *= $p < 0.05$, **= $p < 0.01$, ***= $p < 0.001$.

Supplementary Figure 1

A

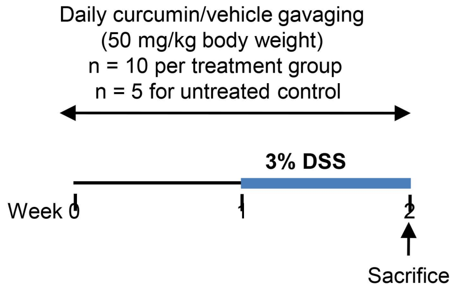

B

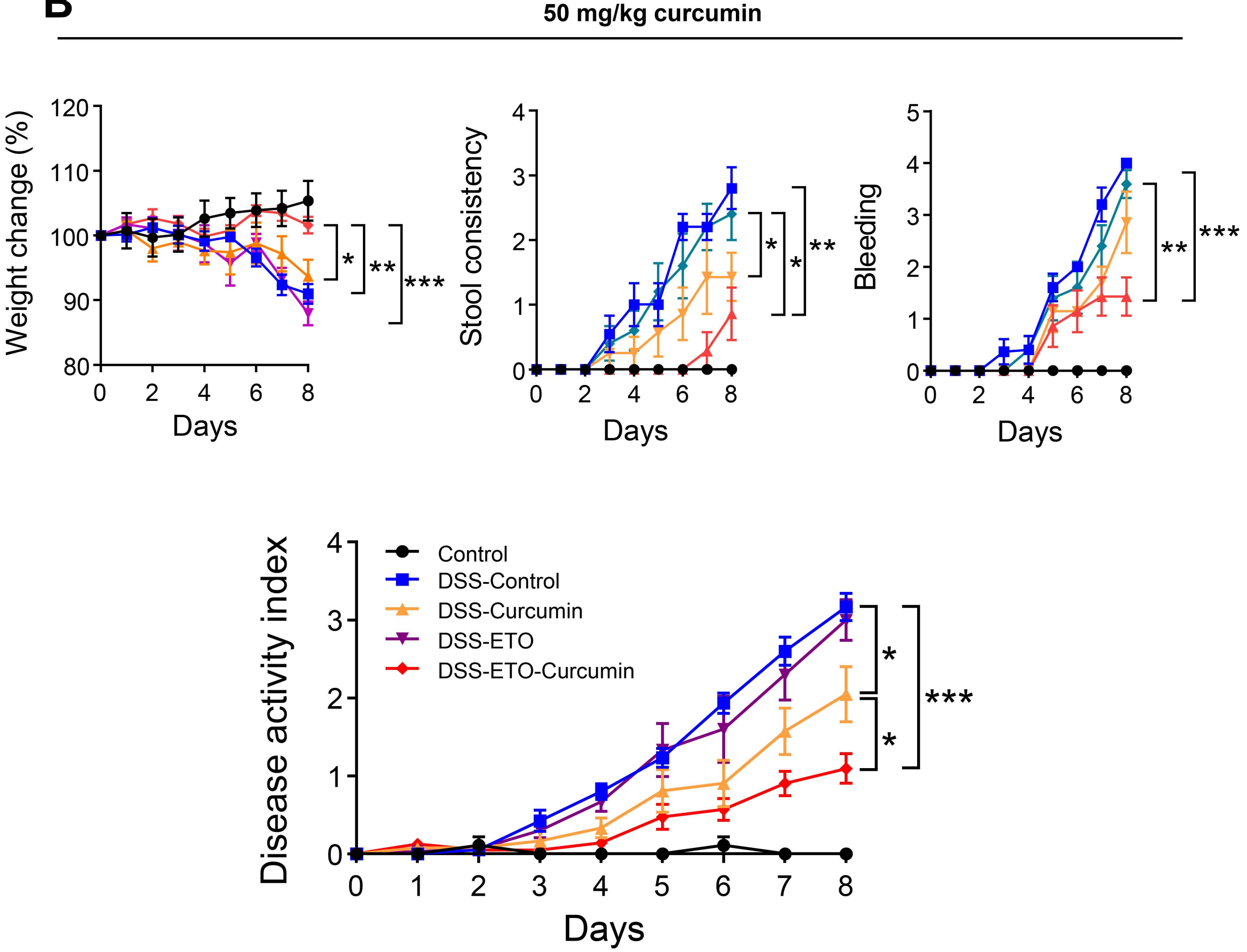

Supplementary Figure 2

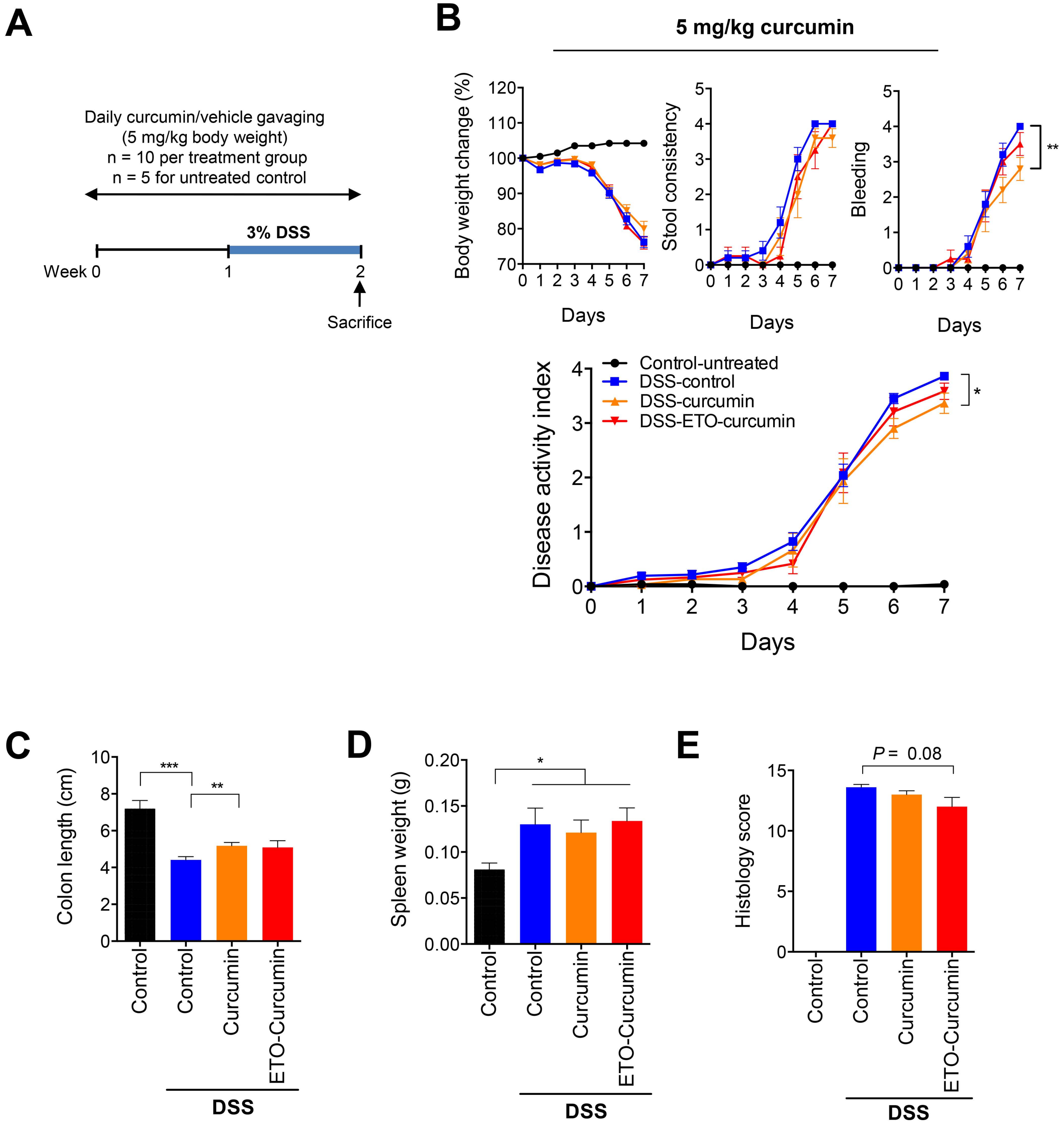

Supplement: Supplementary file 1 — Supplementary methods and figures [file 41598_2017_812_MOESM1_ESM.pdf]
